# Nosocomial infections in ICU comparing HIV infected and not infected patients

**DOI:** 10.1186/1753-6561-5-S6-P78

**Published:** 2011-06-29

**Authors:** AM Costa E Silva, ES Abreu, RDF Feijó, DWCL Santos, F Siroma, CMM Pinto, NJF Cavalcante, R Richtmann

**Affiliations:** 1 Hospitalar Infection Control Comission, Instituto de Infectologia Emilio Ribas, São Paulo, Brazil

## Introduction / objectives

This study describe the epidemiology of nosocomial (NI) notified at ICU in a tertiary level teaching hospital specialized in infectious diseases comparing adults patients HIV infected and not infected

## Methods

From January to December/2009 all patients admitted for more than 48hours at our 17 beds ICU were included. NI definitions were based on the CDC criteria.

## Results

119 NI were notified among 486 patients; the most frequent NI was pneumonia (*n* = 53; 44,5%), followed by primary bloodstream infection (BSI) in 27(22.7%), cardiovascular system – arterial or venous infection (CVS-VASC)(n=16 ;13.4%), urinary tract infection (n=12; 10.1%), intra-abdominal infections (n= 6; 5%), soft tissue/skin infections (n= 4; 3,5%), and surgical site infection (n=1;0.8%). Among pneumonias, ventilator-associated pneumonia were seen in 42 cases (79,2%). Comparing HIV infected and not infected patients, the incidence of VAP in HIV+ was 38.6% X 30.6% HIV-; primary BSI in HIV+ was 22.8% X 22.4% in HIV-; CVS-VASC in HIV+ was 10% X 18.3% in HIV-; urinary tract infection in HIV+ was 12.8% X 6.1% in HIV-; lower respiratory tract infection (non-VAP) in HIV+ was 5,7% X 14,3% in HIV-; intraabdominal infections in HIV+ was 7,1% X 2% in HIV-; soft tissue/ skin infections in HIV+ was 1,4% X 6,1% in HIV-; and surgical site infections in HIV+ was1,4% X zero in HIV-. There was no significant difference comparing groups.

## Conclusion

There were no statistical difference in the topography of NI in HIV infected or not infected at ICU.

## Disclosure of interest

None declared.

